# Beyond Targeted Gene Panels: Whole-Exome Sequencing as a Strategic Platform for Precision Therapeutics in Alzheimer’s Disease

**DOI:** 10.3390/life16091410

**Published:** 2026-08-25

**Authors:** Carlos Perezcano, Mariana Pérez-Coria, Ángel Ricardi-Mendoza

**Affiliations:** Center for Research in Precision Medicine and Clinical Genomics—Genomics 360, Mexico City 01210, Mexico

**Keywords:** Alzheimer’s disease, whole-exome sequencing, integrative neurogenomics, precision medicine, neurodegeneration, neuronal homeostasis

## Abstract

Alzheimer’s disease (AD) continues to be one of the greatest challenges in public health due to its multifactorial and heterogeneous nature, involving multiple physiological axes that encompass a large number of genetic, metabolic, vascular, and inflammatory interactions. In current clinical practice, medical specialties, mainly neurology and psychiatry, still rely on targeted gene panels for genetic evaluation. Although these panels remain effective for certain predefined hypotheses, their restricted and predefined nature limits the detection of the broader spectrum of genetic variation that may contribute to the complex biological interactions underlying neurodegeneration. Whole-exome sequencing (WES) is, from our clinic-based perspective, one of the most comprehensive genomic approaches currently available, since it allows the analysis of the ~19,500 protein-coding regions, and depending on the library approximately 5500 additional clinically relevant genomic loci, including splice sites, untranslated regions, long non-coding RNAs (lncRNAs), pseudogenes, regulatory elements and mitochondrial DNA (mtDNA). It enables the identification of pathogenic variants and variants of uncertain significance (VUS) under the American College of Medical Genetics and Genomics and the Association for Molecular Pathology (ACMG/AMP) classification frameworks. It also expands biological interpretation to variants conventionally classified as benign, which, when interpreted collectively, may contribute to pathway-level contextualization within the hypothesis-generating theoretical framework proposed in this review without implying pathogenicity, causal inference, or immediate clinical actionability. Additionally, WES enables the identification of secondary and incidental findings that may provide clinically relevant information beyond the primary phenotype, thereby supporting preventive surveillance and clinical risk management. This review analyzes the use of WES as a strategic platform for personalized decision-making in contemporary practice given the multifactorial and heterogeneous complexity of AD. It also addresses the complexities and limitations of the ACMG/AMP recommendations for filtering and classification of variants, the lack of standardization between reports and platforms, and the need for physician training, which constitute a great challenge for the translation of data to therapeutic decision-making. While the clinical utility of whole-exome sequencing (WES) in genetic diagnosis and precision medicine is well established, this review additionally proposes a hypothesis-generating theoretical framework whereby variants conventionally classified as benign or of uncertain significance may contribute to pathway-level biological contextualization in Alzheimer’s disease.

## 1. Introduction: Alzheimer’s Disease as a Failure of Neuronal Homeostasis

Alzheimer’s disease (AD) and other related neurodegenerative phenotypes such as frontotemporal dementia (FTD), vascular dementia, Lewy body dementia (LBD), and amyotrophic lateral sclerosis (ALS) continue to represent a growing burden on public health systems with associated expenditures expected to exceed one trillion dollars by 2050 [1]. However, promising research spans anti-amyloid monoclonal antibody therapies, protein-targeting therapies, enzyme inhibitors, mineral-based interventions, functional lifestyle protocols, integrative protocols, and cell and gene therapies, including cellular reprogramming approaches, among others [2,3,4]. Clinically transformative disease-modifying outcomes remain limited and variable across patients [5,6,7,8,9,10] mainly due to the hypothesis that neurodegenerative diseases are multifactorial and heterogeneous, with the involvement of alterations through multiple physiological axes contributing to the loss of neuronal homeostasis [11,12,13], which may partially explain the limited and often variable responses observed with reductionist therapeutic models.

Due to this multifactoriality—which includes alterations in physiological axes such as proteostasis, autophagy–lysosomal function, mitochondrial dysfunction, neuroinflammation, vascular–metabolic regulation, lipid transport, redox and detoxification, synaptic function and excitotoxicity, epigenetics and biological aging, and microbiome–brain interactions [2]—clinical approaches focused on a single therapeutic pathway may provide only partial benefit in biologically heterogeneous neurodegenerative conditions. In addition, many of the current clinical approaches are based on diagnostic imaging methods or biomarkers [14,15] that, although they indicate the presence of neurodegeneration, may provide limited insight into the broader individualized molecular architecture underlying neurodegeneration. Therefore, the use of genomics to gain better insight into individual molecular alterations may provide clinically relevant contextualization. Moreover, some currently used therapies require understanding of pharmacogenomic variability to make informed decisions regarding potential treatment-related interactions associated with specific genetic variants [16,17,18,19,20]. These and many other examples reflect the important clinical paradigm shift that is taking place, moving from one-size-fits-all approaches towards precision medicine [21].

Despite the substantial benefits that genomics has brought to science and medicine, important limitations remain within the field itself, partly due to the evolution of sequencing technologies and partly due to the natural progression of genomic research and association studies, whereby AD is increasingly understood not simply as the consequence of isolated variants such as *APP*, *PSEN1*, and *PSEN2*, but a disease with a complex polygenic architecture involving multiple variants that influence several physiological axes whose biological expression may differ according to each individual genomic and epigenomic architecture [22,23,24,25,26].

As evidence continues to reveal the increasing biological complexity of neurodegeneration, predefined gene panels continue to represent a common strategy in clinical genetic evaluation, potentially limiting broader pathway-level interpretation and longitudinal reinterpretation as genomic knowledge evolves [27,28].

This review examines the potential of whole-exome sequencing (WES) as a strategic platform for precision medicine in AD, discussing its advantages and limitations compared to commercially available gene panels, including the interpretative challenges associated with large-scale genomic datasets and variability across bioinformatic pipelines, reporting systems, and clinical interpretation frameworks.

Unlike previous reviews that primarily focus on the diagnostic applications of WES, pathogenic variants, genome-wide association study (GWAS) findings, or precision medicine in Alzheimer’s disease, the present review additionally proposes a hypothesis-generating theoretical framework in which the expanded genomic information generated by WES may enable the systems-level biological contextualization of variants conventionally classified as benign or of uncertain significance (VUS) according to the American College of Medical Genetics and Genomics and the Association for Molecular Pathology (ACMG/AMP) recommendations, exploring their potential collective contribution to pathway-level neuronal homeostasis in Alzheimer’s disease.

### Targeted Gene Panels in AD: Clinical Utility and Structural Limitations

The clinical genetic evaluation of neurodegenerative disorders has evolved substantially over the last decades, moving from Sanger sequencing approaches focused on single-gene analysis, to targeted PCR-based assays, to next-generation sequencing (NGS) gene panels, to the broader genomic scope of WES and whole-genome sequencing (WGS) [29,30]. Each technological transition also reflected the progressive expansion of knowledge of the genetic architecture involved in AD and related neurodegenerative phenotypes, while also exposing the important structural limitations of the initial techniques based on the selection of predefined genes or single-nucleotide polymorphisms (SNPs). The evolution of genomic sequencing strategies in neurodegenerative disorders is presented in Table 1.

The first segregation studies analyzing AD-associated variants were conducted during the 1990s in families with early-onset Alzheimer’s disease (EOAD), serving as a basis for the identification of genes such as *APP*, *PSEN1* and *PSEN2* with autosomal dominant inheritance [23,31]. These discoveries represented a breakthrough in neurogenetics and established the molecular basis for familial forms of neurodegeneration. Subsequently, candidate-gene studies and genome-wide association studies (GWASs) expanded the genetic landscape of AD with the identification of multiple risk-associated loci, including *APOE*, *TREM2*, *BIN1*, *CLU*, *PICALM* and many other variants involved in lipid metabolism, immune regulation, endosomal trafficking, synaptic integrity, and neuroinflammatory pathways [23]. Recent studies have also suggested a potential association of variants in genes such as *CHI3L1*, *SORL1*, *ABCA7*, *RBKS* and *OR7A10* with biomarkers related to tau accumulation, cognitive performance and structural alterations in neuroimaging studies [32].

Among these, the ε4 haplotype of *APOE* is still considered one of the major common genetic risk factors associated with increased risk of late-onset Alzheimer’s disease (LOAD), where the heterozygous carriers are associated with a two- to threefold increased risk of AD, whereas homozygous carriers may present up to a twelvefold increased risk compared to individuals who do not carry the ε4 haplotype, although these estimates are ancestry-dependent [23,33,34]. However, the contribution of AD-associated variants differs across populations and individuals [35], suggesting that gene panels focused on certain datasets may not capture the broad genomic diversity that underlies neurodegeneration.

As the field has evolved, it has become increasingly evident that sporadic AD does not appear to be explained by isolated variants or strict Mendelian models. The accumulated evidence suggests a much more complex biological landscape in which rare and common variants interact across multiple interconnected physiological axes [22,36,37], potentially contributing through additive and/or modulatory effects to disease susceptibility, progression, and therapeutic response. In this context, gene panels can provide practical utility in certain clinical scenarios, particularly where rapid assessment of a clear genotype–phenotype association is required. However, their predefined architecture may become increasingly restrictive in multifactorial and heterogeneous neurodegenerative phenotypes influenced by complex physiological interactions.

In addition, many of the gene panels currently in clinical use include genes selected according to the evidence available at the time of their development. The genetic field of neurodegeneration is evolving at an unprecedented pace, partly accelerated by advances in artificial intelligence-assisted analytical tools, where new loci, molecular interactions, proteins, and biologically relevant pathways are continuously being identified [38,39,40].

As an illustrative example, a patient clinically diagnosed with frontotemporal dementia (FTD) and behavioral disinhibition underwent a 56-gene dementia panel together with *C9orf72* repeat expansion testing, both of which were reported as negative. Although such findings may exclude several well-established monogenic causes of neurodegeneration, they do not necessarily exclude the potential contribution of additional variants, multilocus interactions, regulatory regions, structural variation, or other biologically relevant mechanisms not captured within restricted targeted approaches [37,39,41].

In this way, sequencing strategies such as WES or WGS (despite their substantially greater bioinformatic complexity) can offer an expanded and reusable genomic substrate for diagnosis and pathway-oriented biological interpretation, as well as longitudinal precision medicine approaches in AD [42].

Rather than representing competing strategies, targeted gene panels, WES, and WGS should be regarded as genomic testing strategies whose optimal use depends on the clinical question, diagnostic objectives, available evidence, and healthcare setting.

## 2. Literature Search Strategy

The literature supporting this narrative review was identified through searches conducted in PubMed, Scopus, and Web of Science from 20 March to 4 August 2026. Search terms were used individually and in combination, including Alzheimer’s disease, whole-exome sequencing, whole-genome sequencing, targeted gene panels, precision medicine, variant interpretation, ACMG/AMP, benign variants, variants of uncertain significance, genome-wide association studies, regulatory variants, neuronal homeostasis, physiological pathways, genetic architecture of Alzheimer’s disease, molecular architecture of neurodegeneration, amyloid-related imaging abnormalities, and anti-amyloid monoclonal antibodies. Peer-reviewed original studies, clinical guidelines, and relevant review articles published in English were considered. Original studies were preferentially cited to support specific biological mechanisms and clinical observations, whereas review articles were used primarily to provide conceptual, historical, and clinical context. Particular emphasis was placed on studies addressing genomic heterogeneity, regulatory and cell-specific effects of genetic variation, pathway-level biological interpretation, and clinically relevant applications of WES in Alzheimer’s disease and related neurodegenerative disorders. Additional references were identified through citation tracking of selected articles. As this work was designed as a narrative review, literature selection and synthesis did not follow a systematic-review protocol or formal meta-analytic procedures.

## 3. Whole-Exome Sequencing: Expanding the Genomic Landscape

### 3.1. Beyond Single-Gene Causality

WES allows the analysis of approximately 19,500 protein-coding genes, and with libraries such as Agilent SureSelect CRE V4, approximately 5500 clinically relevant loci, including splice sites, untranslated regions, long non-coding RNAs (lncRNAs), pseudogenes, regulatory elements, and mitochondrial DNA (mtDNA), may also be analyzed. Depending on the bioinformatic pipeline, WES may also facilitate copy number variant (CNV) detection [43,44,45,46]. This expanded genomic scope of possibilities allows not only a more comprehensive analysis of clearly defined phenotypes but also the filtering and contextualization of additional genes, SNPs, and other genomic variants across physiological axes. It offers the possibility of moving from a single-gene causality perspective to a multilocus architecture that incorporates modifier variants and convergence across biological domains—a true exploration of polygenic complexity.

### 3.2. ACMG/AMP Limitations in Complex Neurodegenerative Disorders

Although the ACMG/AMP recommendations have substantially improved the standardization of filtering and classification of variants in Mendelian disorders, important limitations arise when these guidelines are applied to multifactorial and polygenic neurodegenerative phenotypes such as AD [28]. These recommendations were mainly designed to filter and classify variants using criteria such as population frequency, in silico prediction, functional impact, segregation pattern, de novo occurrence, allelic cis/trans configuration, other reference database information, phenotypic relevance, and comprehensive clinical data [28]. However, in AD and related neurodegenerative disorders, molecular vulnerability may not arise solely from a limited number of variants of different penetrance and expressivity, but may also reflect the cumulative interaction of multiple variants exerting modest individual effects that converge on different physiological axes [28].

Building upon these established limitations, we propose that variants considered benign, likely benign or variants of uncertain significance (VUS) by the ACMG/AMP criteria may still have contextual biological relevance within a broader systems-level biological interpretation [28] and even more so in combination with additional genomic, metabolic, vascular, hormonal, toxic, and exposomic factors [47]. This does not mean that we argue that such variants are reclassified as pathogenic outside the ACMG/AMP classification frameworks, nor that they imply causal inference; we highlight the need for interpretive frameworks capable of contextualizing cumulative pathway-level perturbations beyond strictly binary pathogenic-versus-benign models, particularly in these disorders characterized by complex heterogeneity and incomplete penetrance [28,48,49,50].

### 3.3. Biological Axes and Individualized Physiological Vulnerability

Recent primary studies have demonstrated that the biological consequences of genetic variation are highly dependent on regulatory context, cell type, and molecular networks, providing biological plausibility for systems-level genomic interpretation beyond isolated pathogenic variants [48,49,50]. Nevertheless, the specific framework proposed in this review remains theoretical and requires prospective biological and clinical validation.

**Figure 1:**
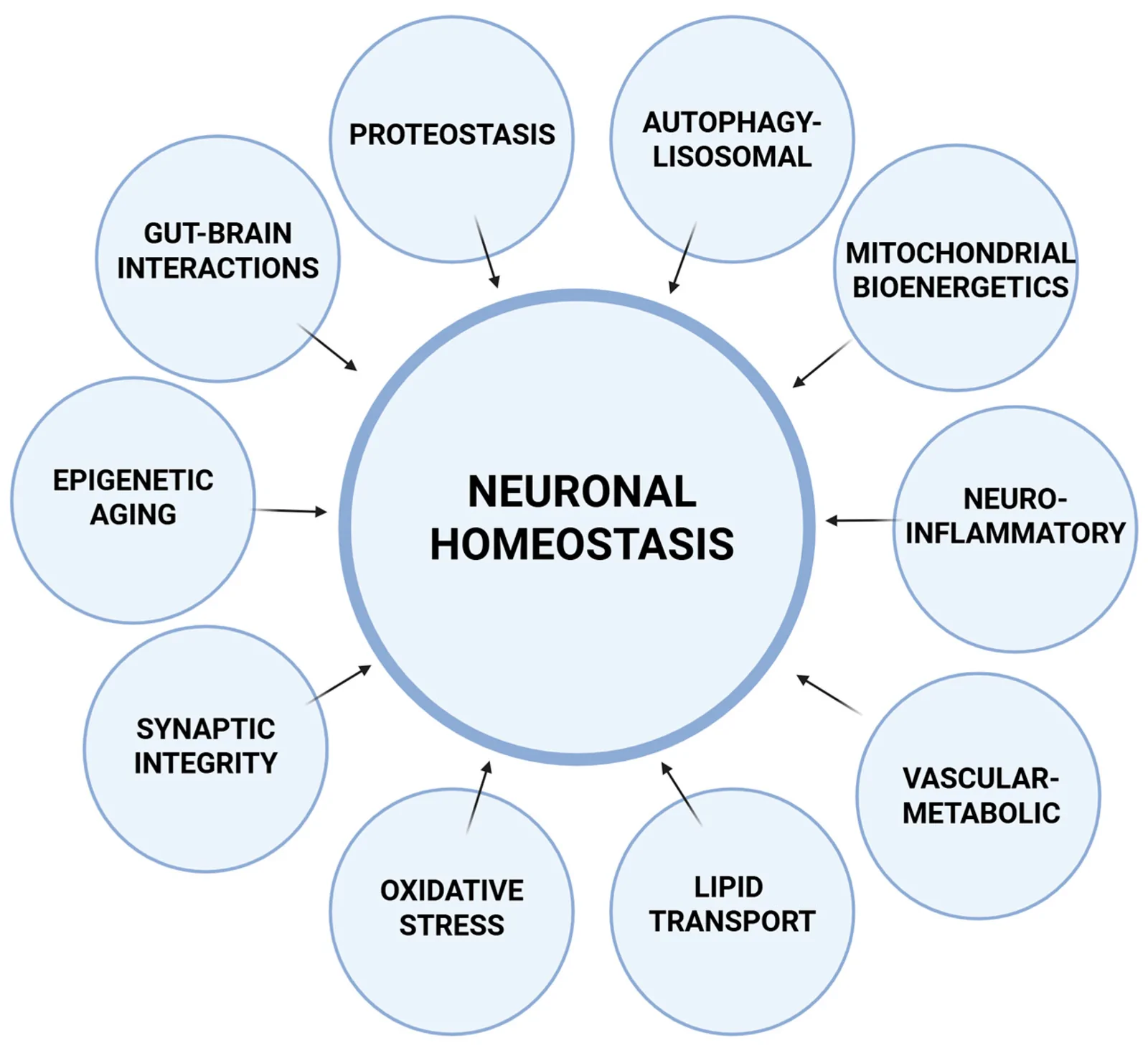
Selected physiological axes contributing to neuronal homeostasis. Conceptual representation of selected physiological domains potentially involved in neuronal homeostasis. Each axis represents a major biological process involved in maintaining neuronal integrity, including proteostasis, mitochondrial function, neuroinflammation, vascular–metabolic regulation, lipid transport, oxidative stress responses, synaptic function, autophagy–lysosomal activity, epigenetic regulation, and gut–brain interactions. Arrows indicate the proposed contribution of each physiological axis to neuronal homeostasis and do not imply a causal relationship. Created in BioRender. PEREZCANO, C. (2026) (https://BioRender.com/mn1cawg, accessed on 21 June 2026).

A growing body of evidence suggests that neurodegenerative disorders may represent the progressive convergence of dysfunction in physiological axes involved in neural homeostasis [13]. These axes include processes related to proteostasis, autophagy–lysosomal function, mitochondrial bioenergetics, neuroinflammation, vascular–metabolic regulation, lipid transport, oxidative stress responses, synaptic integrity, epigenetic aging, and gut–brain interactions (Figure 1). The relative contribution of each axis may differ substantially between individuals, which could contribute to the marked variability in clinical presentation, disease progression, and therapeutic response, and different patterns of comorbidity in patients with AD and neurodegenerative phenotypes [51,52,53,54,55,56,57].

Although ancestry-informed genomic interpretation is still important for the contexts of variant frequency and population risk estimates, each neurodegenerative phenotype appears to require an integrative interpretation beyond population stratification [47].

Within the hypothesis-generating theoretical framework proposed in this review, the comprehensive genomic information generated by WES may facilitate systems-level biological contextualization across multiple physiological axes beyond conventional pathogenicity-oriented interpretation, which primarily restricts genomic interpretation to isolated disease-associated genes.

### 3.4. Secondary and Incidental Findings

Another clinical advantage of WES is the possibility of identifying secondary and incidental findings—following appropriate informed consent—in addition to those of the phenotype-directed approach, which could reveal variants associated with potential cardiovascular, oncological, and metabolic risks, potentially enabling preventive surveillance or modifications in clinical management [58].

Illustrative observations from clinical genomic evaluation of two patients undergoing neuropsychiatric and neurodegenerative assessment revealed secondary findings involving pathogenic variants associated with conditions such as Brugada syndrome (*SCN5A*) [59] and congenital long QT syndrome (*KCNQ1*) [60], respectively, supporting preventive cardiovascular surveillance unrelated to the primary neurological phenotype.

Although secondary and incidental findings remain outside our primary neurodegeneration target, their potential identification contributes to additional longitudinal clinical value compared to other sequencing strategies.

### 3.5. Limitations of WES

Despite the broad diagnostic and clinical interpretative potential provided by WES, it should not be considered as a solution to all genomic questions associated with AD, neurodegeneration or any other phenotype. There are several clinically relevant genomic alterations—repeat expansions, structural rearrangements, intronic variants, CNVs, epigenetic modifications, transcriptomic dysregulation, mitochondrial heteroplasmy—that are not adequately or fully captured by WES [46]. In certain clinical contexts, WGS, transcriptomic, epigenomic, proteomic, and metabolomic analyses may be required for proper biological characterization [61]. In the context of pharmacogenomics, WES cannot replace specialized platforms optimized for the detection of complex star allele architectures, structural variations, or regulatory regions in pharmacogenes [62,63,64].

Additional challenges exist regarding bioinformatics standardization in the filtering and classification of variants, variability across platform outputs, and the translation of complex genomic data into clinically coherent reporting structures and specialist-specific interpretative frameworks [46].

Accordingly, WES should not be regarded as a universal replacement for targeted gene panels, which remain the preferred first-line approach in selected clinical settings where the diagnostic question is well defined and focused genetic testing is appropriate.

For these reasons, WES should not be understood as a definitive standalone diagnostic criterion, but as an efficient genomic platform that can complement additional models of biological interpretation in complex and multifactorial neurodegenerative disorders.

## 4. Precision Therapeutics in AD: Translational Implications and Clinical Integration

The following considerations represent a theoretical framework intended to illustrate how expanded genomic information generated by WES could eventually support future precision medicine strategies following appropriate biological and clinical validation.

Recognition of AD and related neurodegenerative disorders as multifactorial and heterogeneous conditions has progressively increased, which has challenged traditional clinical models based on uniform interventions applied across patients with substantial genotypic and phenotypic diversity [47,51,65]. In this context, within the hypothesis-generating theoretical framework proposed in this review, the expanded genomic characterization offered by WES may contribute to a deeper and more personalized biological contextualization of disease susceptibility, including patterns of physiological vulnerability, potential comorbidities, and therapeutic considerations. Thus, rather than functioning as a tool capable of deterministically predicting treatment response, WES can assist in the biological contextualization of various neurodegenerative phenotypes, potentially offering mechanistically informed clinical reasoning within new precision medicine models [66,67].

One potential future translational implication of expanded genomic information, pending prospective biological and clinical validation, lies in the possibility of biologically stratifying patients according to patterns of physiological vulnerability rather than relying exclusively on symptom-based diagnostic categories. For example, certain patients may manifest a predominance of metabolic vulnerability, whereas others may exhibit vascular and lipid transport alterations, mitochondrial dysfunction, phagocytic dysfunction or neuroinflammatory signaling, among other biological patterns that may influence disease trajectory and therapeutic priorities [51,53,56,57].

The following tables are derived from a single illustrative WES case and are presented solely to demonstrate how the theoretical framework proposed in this review could be applied to pathway-level genomic interpretation. They are not intended to support generalizable biological or clinical conclusions.

Table 2 presents an illustrative example, including ACMG/AMP phenotype-oriented, secondary and incidental findings, as well as variants conventionally classified as benign but contextualized according to their potential pathway-level biological relevance. Although these functional pathways—and particularly their interactions—are not yet fully understood, identifying them may contribute to research-oriented biological contextualization and generate hypotheses for future integrative precision medicine models when interpreted alongside additional biomarkers such as imaging, biochemical, metagenomic and other omics-derived findings [47,65]. Such contextualization should not be used in isolation as a basis for clinical decision-making. The complete genomic annotations are available in Supplementary Material Table S1.

As illustrated in Table 2, under conventional ACMG/AMP-oriented interpretation, variants located in deep intronic regions, untranslated regions, or common polymorphic loci may be appropriately classified as benign or of uncertain clinical significance due to the absence of evidence supporting direct pathogenicity. However, these same variants may still be biologically contextualized according to the physiological pathways in which their corresponding genes participate. For example, *MAPT* is involved in tau-related processes, *ATXN2* in RNA metabolism and cellular stress-response pathways, and *ABCA7* in lipid transport and amyloid-clearance mechanisms [68,69,70]. Although such observations do not imply disease causality, therapeutic actionability, or pathogenic classification, they illustrate how expanded genomic datasets may contribute to a broader pathway-level understanding of heterogeneous neurodegenerative phenotypes.

Precision approaches in AD should not rely exclusively and mainly on pharmacological recommendations. We argue for the design of personalized recommendations in AD that may include nutritional strategies, lifestyle interventions, exercise, sleep, and targeted supplementation approaches that seek to support metabolic, vascular, inflammatory, and oxidative stress-related physiological function, as well as the management of systemic or psychiatric comorbidities [47,76]. Table 3 illustrates precision-oriented integrative considerations across physiological axes.

Many emerging pharmacological therapies require an expanded genomic understanding of each patient’s biological vulnerability. Studies of recent anti-amyloid monoclonal antibodies have highlighted the importance of knowing the patient’s *APOE* status because of the risk of amyloid-related imaging abnormalities (ARIAs) [77,78,79,80,81]. Although this is not the primary objective of WES, it may still provide clinically relevant secondary information in such contexts. This information may support understanding of therapeutic and pharmacological vulnerability (without replacing dedicated pharmacogenomic evaluation) and be able to offer support to make better-informed decisions regarding safety considerations in complex treatments, as is already the case in precision oncology [82,83,84].

Finally, WES may also support longitudinal contextualization of each patient’s genomic vulnerability in relation to comorbidities, since many patients with AD also present or may develop over time cardiovascular, metabolic, psychiatric, and oncological conditions, which influence the disease and treatment tolerance. The reusable nature of WES data may facilitate longitudinal reinterpretation as new phenotypes, comorbidities, genes, and genotype–phenotype associations may emerge over time [62,66].

## 5. Current Challenges in Clinical Implementation

Despite the advantages of WES discussed above, substantial barriers remain to its implementation in clinical practice. One of the most important challenges is the absence of standardized protocols across bioinformatics platforms, laboratories and institutions for genomic interpretation [46]. Differences in sequencing pipelines, annotation tools, filtering and classification strategies, and institution-specific criteria regarding which findings should be reported and how they should be communicated may generate considerable inter-report variability [85]. This issue is particularly relevant in multifactorial and heterogeneous phenotypes such as AD, where biological interpretation may extend beyond conventional ACMG/AMP classification frameworks [28].

The immense complexity of genomic data also represents a substantial interpretative burden for clinical geneticists and treating physicians. Although WES allows the identification of a large number of potentially relevant variants, determining their biological significance in heterogeneous neurodegenerative phenotypes continues to represent a major interpretative challenge, particularly when these findings are integrated with additional biomarkers and omics-derived datasets [86]. ACMG/AMP filtering and classification criteria also remain limited when it comes to contextualizing pathway-level genomic vulnerability across physiological axes, which may collectively contribute to phenotypic manifestation.

Another important challenge in clinical translation involves educational programs in medical training and multidisciplinary integration. Many physicians, geneticists, and even molecular biologists have received limited formal training in genomics, bioinformatics, and precision medicine, particularly regarding systems-level biological interpretation [87]. Consequently, the translation of a large amount of complex laboratory and bioinformatics data into clinical decision-making currently requires collaboration among several specialists involving neurologists, psychiatrists, geneticists, bioinformaticians, and potentially specialists in precision medicine and clinical genomics. This approach may involve interdisciplinary consultations or, ideally, multidisciplinary teams (MDTs), which are already more extensively implemented in oncology [88,89].

Another major barrier to implementation involves costs and logistics. Although the prices of NGS analysis continue to decrease as genomic technologies become more accessible, wet-laboratory infrastructure, bioinformatics, data storage, analysis, reanalysis, and multidisciplinary interpretation require substantial investment in equipment and specialized personnel [90]. In addition, insurance coverage for genetic testing remains limited and is highly variable across health systems, potentially limiting equitable access to precision medicine approaches in AD and related neurodegenerative disorders [64].

The implementation of sequencing strategies also raises ethical considerations involving informed consent, data protection and management, clinical records, reporting of secondary and incidental findings unrelated to the primary phenotype, psychological impact, and family implications [91]. Furthermore, the potential for longitudinal reinterpretation of WES also introduces additional ethical and operational challenges concerning the management and communication of newly reinterpreted findings [92,93].

Finally, despite the growth and advances in precision medicine applied to AD, many proposed hypotheses regarding pathway-level genomic influence still lack robust prospective validation in large longitudinal cohorts [40].

In summary, the clinical utility of WES is well established; however, its use for pathway-level biological contextualization within the framework proposed here should currently be considered research-oriented rather than a deterministic predictor of disease trajectory or therapeutic response. This interpretation is intended to generate biological hypotheses and should not be used in isolation as a basis for clinical decision-making. Validation of the theoretical framework proposed in this review will require prospective longitudinal cohorts integrating WES with multimodal biological and clinical data. Evidence supporting its clinical utility should demonstrate reproducible pathway-level biological contextualization, improved biological patient stratification, and incremental predictive value for disease progression or therapeutic response beyond conventional interpretation based exclusively on variant pathogenicity. Such evidence, together with standardized results and interdisciplinary collaboration, will be essential for the responsible clinical implementation of the proposed pathway-level biological contextualization framework applied to comprehensive genomic data generated by WES or WGS in precision medicine for AD and other neurodegenerative disorders.

## 6. Future Directions

As technology advances, precision medicine applied to neurodegenerative disorders, and increasingly across medical specialties, is gradually incorporating the convergence of genomics with additional biological layers including biochemistry, advanced neuroimaging, plasma biomarkers, epigenomics, transcriptomics, proteomics, and metabolomics, as well as microbiome-derived information [15,47]. In this rapidly evolving field, the clinical value of genomics will not depend solely on variant detection, but on its ability to contextualize genomic findings within multidimensional and interdisciplinary models that address complex neuronal vulnerability states and disease progression [94].

The parallel incorporation of WGS and emerging omics technologies may further expand the biological resolution achieved through WES. WGS enables characterization of non-coding regions, promoter and regulatory elements, and structural variation, whereas complementary omics approaches—including epigenomics, transcriptomics, proteomics, metabolomics, and microbiome functional profiling—provide additional functional layers that may contribute to a more dynamic characterization of biological interaction networks involved in AD and neurodegeneration [95].

In this context, the incorporation of artificial intelligence, both in filtering/classification processes and in the development of advanced integrative bioinformatics interpretative frameworks, will be important for managing, analyzing, and contextually interpreting the large number of multi-omics findings, as well as their potential interactions across biological layers and physiological axes [40,96,97,98,99]. Machine learning models may contribute to the improved identification of complex pathway interactions, support integrated clinical reasoning, and identify potential therapeutic response patterns and individual risk trajectories, which are currently difficult to implement with traditional clinical strategies [100].

Recent translational efforts already applied in clinical settings propose integrative neurogenomic workflows for neurodegenerative disorders that illustrate the advantages and limitations of the application of multidimensional models of precision medicine that include genomics, biochemistry, pharmacogenomics, epigenomics, and metagenomics of the gut microbiome, which through MDT-based interpretation may facilitate personalized clinical contextualization. These models are in their early stages and require longitudinal validation, but they may represent the beginning of future evolution toward individualized and mechanistically informed therapeutic strategies applied to AD and related neurodegenerative disorders [47].

Finally, emerging cell and gene therapy strategies highlight the importance of individualized genomic interpretation in neurodegenerative medicine. Allele-specific modulation of *APOE*-related pathways, antisense oligonucleotides (ASOs), gene-silencing technologies, neural progenitor cell transplantation, induced pluripotent stem cell (iPSC)-derived regenerative therapies, and exploratory partial cellular reprogramming strategies are increasingly being investigated across neurodegenerative disorders [101]. Regulatory advances in Japan have recently approved the use in humans of the first products derived from cell reprogramming that illustrate the accelerating translation of regenerative medicine strategies from laboratory research into clinical application [102].

However, many of these interventions remain experimental and continue to face ethical, biological, and therapeutic efficacy challenges, including tumorigenicity, immune compatibility, delivery constraints, off-target effects, epigenetic instability, long-term safety uncertainty, and incomplete biological control [103]. Even if successful delivery is achieved, the restoration of neuronal homeostasis cannot necessarily be guaranteed; silencing *APOE4*, degrading amyloid beta, reducing tau pathology, replacing neuronal populations, delivering progenitors and ASOs, and editing genomic sequences through clustered regularly interspaced short palindromic repeats (CRISPR)-based strategies in key genes do not necessarily imply the restoration of neuronal circuits, vascular integrity, phagocytic function, proteostasis, metabolism, glial function, inflammation, synaptic function, and network-level dynamics required to sustain neuronal homeostasis [86,104,105,106,107,108].

Consequently, all this biological complexity reinforces the importance of incorporating individualized therapeutic frameworks capable of integrating genomics and multidimensional data from each patient, which may contribute to the development and optimization of future therapeutic strategies.

## 7. Conclusions

Alzheimer’s disease and related neurodegenerative disorders appear to emerge more from multifactorial and heterogeneous biological architectures involving the convergence of several physiological axes than from isolated pathogenic mechanisms. In this context, WES represents a broad and reusable genomic analysis platform, capable of providing pathway-oriented biological contextualization across physiological domains, longitudinal reinterpretation, and a more individualized and informed approach to precision medicine, extending beyond the restrictive scope of predefined gene panels.

Rather than functioning as a deterministic predictor of disease trajectory or therapeutic response, WES facilitates multilocus genomic interpretation (including the possibility of longitudinally analyzing various clinical phenotypes and comorbidities using the same genomic dataset), supporting emerging integrative precision medicine models. Although there are substantial challenges in clinical implementation—such as standardization of variant filtering and classification, clinical interpretation frameworks, economic accessibility, and bioinformatics complexity—its advantages position WES as a robust and efficient genomic platform that contributes to the future development of individualized and mechanistically informed approaches for AD and related neurodegenerative disorders, while paving the way for the future integration of broader genomic and multi-omics technologies.

## Figures and Tables

**Table 1:** Evolution of genomic sequencing strategies in neurodegenerative disorders.

Approach	Genomic Scope	Main Clinical Utility	Interpretative Potential	Limitations
Sanger Sequencing	Single genes or selected exons	Confirmation of highly penetrant pathogenic variants	Focused monogenic interpretation	Limited scalability; low throughput; unsuitable for complex polygenic architectures
Targeted PCR-Based Panels	Small predefined groups of genes or variants	Focused, rapid, and clinically interpretable evaluation of established disease-associated loci	Restricted to predefined hypotheses	Limited pathway coverage; static architecture; inability to capture broader genomic complexity
NGS Targeted Panels	Expanded predefined gene sets	Broad yet hypothesis-driven evaluation of multiple neurodegeneration-associated genes	Improved detection of known pathogenic and risk-associated variants	May exclude modifier variants, non-targeted loci, and evolving pathway interactions
Whole-Exome Sequencing (WES)	~19,500 coding genes and clinically relevant coding-associated loci	Broader genomic evaluation across heterogeneous phenotypes	Pathway-oriented interpretation; longitudinal reinterpretation; multilocus contextualization	Limited detection of structural variants, deep intronic alterations, repeat expansions, and regulatory elements; limited determination of variant phase and incomplete coverage of some exons.
Whole-Genome Sequencing (WGS)	Coding and non-coding genome-wide analysis	Comprehensive genomic characterization	Expanded biological resolution including regulatory and structural variation	Higher resource requirements, greater bioinformatic complexity, interpretative burden, and uncertain clinical significance of many findings

**Table 2:** Illustrative WES findings and pathway-level biological contextualization in a neurodegenerative phenotype.

Type of Finding	Gene Variant/rsID	ACMG/AMP Classification	Biological Axis	Potential Clinical Relevance
Phenotype-oriented	*MAPT*	VUS	Proteostasis	Potential contribution to neurodegeneration-related protein aggregation pathways [68]
Phenotype-oriented	*ATXN2*; rs7969300	Benign	Proteostasis	Potential relevance to RNA metabolism and cellular stress-response pathways [69]
Phenotype-oriented	*ABCA7*; rs3752246	Benign	Lipid transport	Potential contribution to lipid transport and amyloid-clearance biology [70]
Phenotype-oriented	*GBA1*; rs2230288	Benign	Autophagy–lysosomal function	Potential relevance to lysosomal function and cellular homeostasis [71]
Phenotype-oriented	*APOE*; rs440446	Benign	Lipid transport	*APOE* regulatory variant associated with lipid transport, neuronal repair, and cognitive aging [72]
Phenotype-oriented	*CD33*; rs12459419	Benign	Neuroinflammation	Potential relevance to microglial activation and phagocytic pathways [73]
Incidental	*CYP1B1*; rs957253424	VUS	Oxidative stress responses	Potential relevance to xenobiotic metabolism, oxidative stress regulation, steroid metabolism, and cellular detoxification pathways; increased susceptibility to the development of primary open-angle glaucoma (POAG) [74,75]

(1) No pathogenic phenotype-oriented variants were identified according to ACMG/AMP classification criteria, and no secondary findings were identified based on the ACMG SF v3.3 recommendations list. (2) Complete genomic annotations are available in the Supplementary Material. (3) Biological axis assignment and integrative considerations are based on the known biological functions of the corresponding genes and are intended exclusively for exploratory pathway-level contextualization. They should not be interpreted as evidence of pathogenicity, causality, or therapeutic actionability of the specific variants identified.

**Table 3:** Illustrative precision-oriented integrative considerations across physiological axes in neurodegeneration based on WES.

Biological Axis	Genomic Context	Illustrative Integrative Considerations
Proteostasis	*ATXN2*, *MAPT*	Precision nutrition, exercise, sleep optimization, reduction in chronic inflammatory burden
Autophagy–lysosomal function	*GBA1*	Promote physical activity, metabolic health, and interventions supporting lysosomal and cellular recycling pathways while minimizing mitochondrial stressors
Mitochondrial bioenergetics	No relevant variants identified in this illustrative case	No relevant variants identified in this illustrative case
Neuroinflammatory	*CD33*	Optimization of sleep quality, maintenance of metabolic health, regular physical activity, and other interventions known to modulate chronic neuroinflammation and microglial activation
Vascular–metabolic regulation	*APOE*	Management of vascular risk factors, insulin resistance, dyslipidemia, and cardiovascular health
Lipid transport	*APOE*, *ABCA7*	Optimize lipid metabolism, cardiovascular health, sleep quality, glycemic control, and anti-inflammatory dietary patterns
Oxidative stress responses	*CYP1B1*	Minimize environmental toxicant exposure, optimize antioxidant status, support detoxification pathways through nutrition
Synaptic integrity	No relevant variants identified in this illustrative case	No relevant variants identified in this illustrative case
Epigenetic aging	No relevant variants identified in this illustrative case	No relevant variants identified in this illustrative case
Gut–brain interactions	No relevant variants identified in this illustrative case	No relevant variants identified in this illustrative case

Illustrative integrative considerations are intended exclusively as examples of pathway-oriented contextualization and should not be interpreted as evidence-based clinical recommendations. Clinical decision-making requires integration with additional clinical, biochemical, imaging, and other relevant patient-specific information.

## Data Availability

No new data were created or analyzed in this study. Data sharing is not applicable to this article.

## Supplementary Materials

The following supporting information can be downloaded at https://www.mdpi.com/article/10.3390/life16091410/s1. Table S1: Complete WES Genomic Variant Annotations in a Neurodegenerative Phenotype.
